# An institutional ethnography of chronic pain management in family medicine (COPE) study protocol

**DOI:** 10.1186/s12913-015-1078-7

**Published:** 2015-11-05

**Authors:** Fiona Webster, Onil Bhattacharyya, Aileen Davis, Rick Glazier, Joel Katz, Paul Krueger, Ross Upshur, Albert Yee, Lynn Wilson

**Affiliations:** Department of Family and Community Medicine, Institute of Health Policy, Management and Evaluation, University of Toronto, 500 University Avenue, 5th Floor, Toronto, ON M5G 1V7 Canada; Women’s College Research Institute, 790 Bay St, 7th Floor, Toronto, ON M5G 1N8 Canada; Division of Health Care and Outcomes Research, Toronto Western Research Institute, University Health Network, University of Toronto, Toronto, Canada; Institute of Health Policy, Management and Evaluation, Departments of Physical Therapy and Rehabilitation Science Institute, University of Toronto, 399 Bathurst Street, MP11-322, Toronto, ON M5T 2S8 Canada; ICES Central, Primary Care & Population Health Research Program, G1 06, 2075 Bayview Avenue, Toronto, ON M4N 3M5 Canada; Department of Psychology, York University, 4700 Keele St., BSB 232, Toronto, ON M3J 1P3 Canada; Department of Anesthesia and Pain Management, Toronto General Hospital, 200 Elizabeth St., 3EB-317, Toronto, ON M5G 2C4 Canada; Dalla Lana Faculty of Public Health, 155 College Street, Toronto, ON M5G 1L4 Canada; Sunnybrook Health Sciences Centre, 2075 Bayview Ave., Room MG 371B, Toronto, ON M4N 3M5 Canada

**Keywords:** Complex patients, institutional ethnography, chronic pain

## Abstract

**Background:**

Patients with chronic conditions and multiple comorbidities represent a growing challenge for health care globally. Improved coordination of care is considered essential for providing more effective and cost-efficient care for these patients with complex needs. Osteoarthritis is one of the most common and debilitating chronic conditions, is the most frequent cause of chronic pain yet osteoarthritis care is often poorly-coordinated. Primary care is usually the first contact for patients requiring relief from chronic pain. Our previous work suggests discordance between the policy goals of improving patient care and the experience of osteoarthritis patients. We plan to investigate the empirical context of the primary care setting by focusing on primary physicians’ conceptualizations and performance of their work in treating complex patients with chronic pain. This will allow for an exploration of how primary health care is – or could be – integrated with other services that play an important role in health care delivery.

**Methods:**

Our study is an Institutional Ethnography of pain management in family medicine, to be carried out in three phases over 3 years from 2014/15 to 2018. Over the first year we will undertake approximately 80 key informant interviews with primary care physicians, other health care providers, policymakers and clinical experts. In the second year we will focus on mobilizing our networks from year one to assist in the collection of key texts which shape the current context of care. These texts will be analyzed by the research team. In the final year of the study we will focus on synthesizing our findings in order to map the social relations informing care. As is standard and optimal in qualitative research, analysis will be concurrent with data collection.

**Discussion:**

Our study will allow us to identify how the work of coordinating care across multiple settings is accomplished, in practice as well as discursively and textually. Ultimately, we will identify links between everyday experience of care for patients with chronic pain, and broader discourses related to health care system inefficiencies, integration and patient-centred care. An expected outcome of this study will be the development of new, or augmentation of existing, models of care, that are based in the local realities of primary care practice.

## Background

Providing cost-effective care for the increasing numbers of complex elderly and/or co-morbid patients is a global problem facing industrialized and non-industrialized nations [1]. With an aging population and individuals living longer lives, healthcare costs continue to rise at rates faster than other parts of the economy [2]. In response, payers have sought to contain spending growth through healthcare rationing and efficiency gains. Exacerbating these challenges are the growing numbers of individuals with chronic disease and multi- morbidities, a proportion of who develop complex health conditions. For example, in Ontario, one study estimated that complex patients constitute 1 % of the province’s 13.7 million people but accounted for 34 % of Ontario health care expenditures [3]. Similar research has identified the same trend from the United States [4] and the United Kingdom and Europe [5]. Costs associated with managing complex care patients have generated a growing sense of urgency due to the increasing emphasis on accountability, the sustainability of health care, and pressures posed by population aging.

In many jurisdictions across Canada, governments have launched priority initiatives to improve the care costs of complex patients. These initiatives have been based on the assumption that improved coordination will lead to faster care at reduced costs [6]. Often these initiatives have called for researchers to develop innovative models that are both patient-centred and cost effective. Moreover, in Canada, as well as in many other countries, one of the main problems identified in the care of complex patients is poor coordination of services [1]. Poor coordination of services has been described as impeding both good care and efficient use of health care dollars. It has been estimated that in the province of Ontario better integration of care would result in savings of $4 to $6 billion per year from reductions in redundant services, improved coordination and provision of more appropriate services [2].

At the same time that Ministries of Health, health services researchers and media are recommending cost-saving measures and creation of new and innovative models of care, those working in social science have been questioning the nature and framing of these public policy debates. Many have challenged the hidden assumptions underlying the import and application of performance measurement and accountability principles developed in business settings into health care settings [7–9] and have sought to draw attention to the implications of the resultant discourses that are shaping public policy debates. They have, for example, challenged the “virtual realities” created by systems of accounting based on administrative databases [7–9] that produce this notion of “high users” of health care resources cited above. Nevertheless, these debates are often not well- integrated into mainstream conversations about health policy and practice [10].

In this context, our study builds on an innovative Canadian Institutes of Health Research (CIHR) funded research program that uses a social science lens through which to view the organization of the care of patients with chronic osteoarthritis (OA) pain, many of whom have multiple co-morbidities [11]. It is clear that that there is a need for such research as this is where the majority of patient care takes place and is seen as a key determinant in complex patients’ need for care [12]. Moreover, this research also seeks to explore how other stakeholders are involved in coordination of care not as separate objects of study but as part of the same patient care “system”. This will be accomplished by speaking with primary care physicians and asking them to describe both what they do and who else is involved in the care they deliver (for example, pharmacy, orthopedic surgeons, physiotherapists) to patients with multi- morbidities and chronic pain.

Complex patients are the focus of much debate and concern in the contemporary healthcare context. They are defined as those who have more than one of the following five major chronic conditions: arthritis, diabetes, heart disease, chronic lower respiratory tract disease and stroke [13]. Patients with these chronic conditions may also suffer from mental health problems or addictions [13]. Multi- morbidity is even more important as each condition may influence the care of the other condition(s), result in interactions between therapies and/or direct contraindications to therapy and thereby limit life expectancy [14]. At the patient level, multi-morbidity has been found to affect quality of life, ability to work, disability and mortality [15]. In addition, the process of navigating care across different specialists is burdensome for patients [16] and is a patient safety issue [3]. Our previous work in patients with osteoarthritis (OA) pain suggests discordance between the policy goals of improving patient care and the experience of patients [17, 18].

Arthritis, specifically OA, is one of the most common, disabling, and costly chronic diseases [19–21]. It is a degenerative joint disease that most commonly affects the knees, hips, hands, and spine and is characterized by a slow evolution of symptoms (joint pain and stiffness) and disability over time [22–24]. Arthritis is the most frequent cause of chronic pain which is debilitating to the individual and extremely costly to society. Primary care is most often the first contact for people seeking symptom relief. Truly interprofessional care (IPC) is necessary to provide appropriate evidence-based pain management and self-management support for these patients who are seeking appropriate strategies to ameliorate and limit progression of their chronic pain and resultant disability. Yet achieving successful IPC often remains an elusive goal in practice [25].

Several research gaps have been identified in relation to primary care in musculoskeletal care, including OA [26]. These include well-known issues such as a lack of comprehensive management that includes exercise and weight loss strategies [27], a focus on other chronic conditions or co- morbidities that may be considered more urgent [28, 29], and low referrals rates to both physiotherapy and total joint arthroplasty [29]. There are also ongoing controversies associated with the use of opioid medication to manage pain and concerns with possible medication dependence, misuse, and addiction [30–32]. Finally, at the patient level, there are many socio-economic factors that influence help-seeking, referral, and treatment, with some patients “falling through the cracks” [33]. However, despite our knowledge of these various gaps, what remains unknown are strategies or models of care that, while rooted in primary care – the “medical home” for most patients [34] – are coordinated effectively with other care providers, policymakers, patients, and their families and offer the benefits of IPC.

Primary care is the context for most OA education and management. The role of context in health care has been increasingly recognized as important at the local level [35]; however, context is often described in abstract terms and may refer only to a description of geography or place [36]. The need to investigate the empirical context of the primary care setting, not as it exists in isolation or in the abstract, but as it pertains to other providers in the patient journey is critical in order to improve patient care of OA pain management. This will allow for an exploration of how primary health care is – or could be better– integrated with other services that play an important role in health, such as housing, education, and income. The patient-centred medical home has been introduced as a chronic care model that can reduce costs [37]. It emphasizes the need for “care management” to be based in primary care and has become increasingly important in Canada as well as internationally [38]. Several studies of care management in primary care show convincing evidence of improving quality [39–45]. These studies measured a variety of quality outcomes, including patient satisfaction, functional ability [capacity to perform basic activities of daily living], mortality, bed disability days and overall quality of life. The results of care management studies in primary care are mixed regarding reductions in hospital use and healthcare costs. All of these studies enrolled patients with multiple chronic conditions who were at high or moderate risk of incurring major health care costs. Each program placed substantial emphasis on training the care manager team, keeping care manager panel sizes at reasonable levels, forging a close relationship between care managers and primary care physicians and including care manager interactions with patients in-clinic, and at home by telephone.

This study will employ the approach of institutional ethnography (IE) to investigate how primary care physicians define, encounter and manage complex patients who experience chronic pain. Our overarching research question is: How do primary care physicians describe the *work* they do in caring for patients with complex chronic pain conditions? This question will become our starting point, rather than the end point, to allow our team to explicate how care is put together at the level of the institution (macro) with attention to how care is coordinated in local practice (micro). Using an expanded definition of *work* will allow us to understand the many types of work that physicians do (e.g. examining patients, making phone calls, filling out forms, email) and also alerts us to how the discourses and concerns of institutions enter into the everyday language of care providers. This will achieve the following specific objectives:

1. Describe carefully and empirically the work that goes into caring for complex patients with chronic pain and multi-morbidities beginning from the standpoint of primary care physicians and working outward to capture the often invisible social relations informing patient care;
2. Follow in each setting the textual, informal and formal practices implemented by the providers, as their work is coordinated with that of others in different settings. This will create a “map” of the social relations of primary care provision of OA chronic pain management that includes the perspectives of all key stakeholders and will allow for the creation/augmentation of new models of care that are effective, efficient and sustainable, ultimately improving the patient’s experience of CARE.
3. Provide a link between everyday experience to broader discourses related to health care system inefficiencies, integration and patient-centred care. These links will be the mechanism by which findings from our work can be “ replicated” and applied to other provinces and internationally.

## Methods

Institutional ethnography (IE) was developed by sociologist Dorothy Smith [46–50]. According to Smith, the social strategy she developed is “constrained by the project of creating a way of seeing, from where we actually live, in the powers, processes and relations that organize and determine the everyday context of that seeing” [50]. IE refers to an approach to inquiry rather than a set of methods. It uses people’s everyday experiences as the starting point for an exploration of the often invisible social relations that underpin or organize their experiences [47–50]. Based on Smith’s understanding of the social organization of knowledge, it allows for an examination of the complex social relations organizing people’s experiences of their everyday working lives. IE makes use of several types of data, typically including interviews, observations and texts. However, IE studies may differ in the extent to which they employ the various data collection strategies [50]. In this study we will rely on interviews and texts and also observations when possible. Ethics approval for this study was obtained through the University of Toronto Research Ethics Board.

### Sampling and recruitment (standpoint): year one

Understanding the social world requires taking up a specific position as a starting point from which to begin to explore how things are put together the way that they are [50]. In this sense, IE is sampling an institutional process rather than a population. All qualitative sampling is purposive rather than experimental and there are multiple purposive sampling techniques [55]. The goal is not to be representative of the broader population, but rather to understand a phenomenon or process in-depth.

In practical terms, our study will include any primary care physicians working in community and academic hospitals and in any type of primary care organization, such as family health teams, solo practice, and Community Health Centres. We will purposively select family physicians from each type of practice model within 4 Local Health Integrated Networks (LHINs), one northern, one southwestern, one downtown Toronto and one eastern, and within each we will select academic and community hospitals. Participants will initially be identified through members of our team [who in turn are linked to other collaborators across different sites] as we embark on our study. Following REB approval, the principal investigator will send out an email with a letter of information and a consent form requesting that the recipient participate in an interview in a location of their choosing and at a date and time convenient to them. At the conclusion of each interview, participants will be asked to suggest other potential participants whom they identify as being involved in the coordination of their working lives (for example a hospital administrator or site supervisor).

In an IE study, sampling, while purposive, does not follow standard strategies such as maximum variation or theoretical sampling [55]. The identification of research sites, informants and texts cannot be pre-determined but proceeds through the process of inquiry [53]. For example, primary care physicians refer patients for joint replacement surgery and then care for them post-surgery. We might therefore speak to those involved in the referral and repatriation process, including orthopedic surgeons and pharmacists. This is known as snowball sampling [55]. IE researchers follow sequences of action, with one informant’s interview leading the way to the next or to a text for analysis.

### First level data collection: interviews

We estimate that we will conduct approximately 80 interviews over 18 months. Estimates of qualitative interviews are classically difficult to determine prior to entering the field given the exploratory nature of the IE approach. Therefore, our estimate is based on presupposing that at a minimum we might interview 5 primary care physicians across 4 different sites and/or settings (=20 participants). We may interview a greater or lesser number of these participants depending on what they share with us, we might interview the same informant twice, and may also interview others who we have yet to identify as playing a key role in the social coordination of chronic pain management. In addition and as previously noted, IE researchers look for something outside of the experiences of key informants which is largely invisible to them and yet that enters into and coordinates their work with those of others of whom they ma y not even be aware. Exploring relations beyond primary care physicians’ experience means learning from others who work in related settings. Hence, we will also interview other health care providers, policymakers and patients, and their family members. These interviews will take place in a location that is convenient for the participants (i.e. workplace, community setting or home) and formal written consent will be obtained.

The content of our in-depth interviews will be guided by an emphasis on the actual work that people perform. Most research-based descriptions of physician decision-making are abstract accounts that are not connected to the actual work they engage in [34] or refer to individual psychological explanations for physician behavior [12, 35, 36]. This concept of work and of work knowledge is what the ethnographer draws on in talking to informants and will comprise the content of the interviews. This also opens the possibility of exploring informal aspects of work that are rarely accounted for in professional practice guidelines and of analyzing the role of texts and writing in the work physicians and policymakers perform.

### Analysis

The procedural steps involved in analyzing data are similar to that practiced in other qualitative research (QR) approaches. Data is transcribed and coded so that it can be analyzed. Codes identify features of the data that are pertinent to the research questions and organize data into more concise ideas that can be eventually grouped into topics. They are often recurrent keywords or concepts that are supported by interview data (i.e. quotes). Coding of the first few transcripts will be performed by the P.I., the interviewer and another member of the research team. Multiple coding is a useful way of developing reflexivity rather than a tool to confirm the “truth” of the data [57]. Reflexivity used in this way refers to the process by which we critically examine our own assumptions about the world [57]. Through comparison of our developing understanding of the transcript data, each member of the coding team has an opportunity to check their own assumptions and ideas. The closest equivalent for this type of analytic technique would be thematic analysis [58, 59]. However, codes as they are used in IE are not used to develop themes but will reflect our analytic interest in explicating how the work that is performed by an actor in one situation (locally) is coordinated extra- locally. Finally, we will also hold team meetings in which we will discuss and reflect on our emerging understanding of what is being described. The thoughts and comments that result from all meetings will be recorded as extensive marginal notes throughout the interviewing, transcription, and coding phases, with the goal of focusing thoughts around the emerging concepts. These meeting notes also become part of the audit trail in which the team keeps careful track of all interpretative and theoretical decisions that are made about the phenomena being studied.

### Second-level data collection: year two

#### Text and discourse analysis

Through interviews and also drawing on our own institutional knowledge, our team will identify a series of texts and discourses that are present in the language of participants as they describe their everyday work practices in caring for complex patients. Texts refer to any document that has a fixed and replicable character (such as a care pathway) and includes any documents that can be become distributed and subsequently used by users in different places and at different points in time. Texts are “activated” when they are read, completed or filled in (i.e. discharge forms). Higher order texts [52] are those that are not often visible in local settings but become active in the actual settings of people’s work. An example of this would be the Wait Times Strategy. These higher order texts do not rule by prescription but by establishing the “concepts and categories of which what is done can be recognized as an instance of expression of the textually authorized procedure” [54]. Texts then both standardize and mediate social relations [52].

Our interview data will be analyzed for “clues” that point us to texts, which may help us explain organizational details missing from experiential accounts. The genre of text that participants identify cannot be pre-determined but may include policy documents, related to different sectors, such as education or health or clinical documents such as care pathways. For example, an interviewee may make passing mention of “filling out Form X in order to accomplish Y,” which we would then follow up by tracking down Form X and any associated governing policies [38]. Tracking a document across various sites helps to create a social “map” of the various work processes involved in coordinating a particular model of care. The documents we seek will be publicly available texts. We will assemble the various documents that may play a role in care coordination between primary care physicians and other professionals and across sites in order to better understand the individual accounts being provided to us. Our preliminary work has led us to identify the several key documents as being central to our analysis but others will be added during the course of our interview.

Textual analysis will also include analyses of relevant discourses. Our use of the term discourse draws on the idea that language is active; that is, it has a constitutive function as well as a descriptive one [46]. In our work we will analyze the use of several terms in everyday language used by physicians, other clinicians and policymakers in order to better ask: What is left out by the use of these terms? Who benefits from these terms? What is rendered invisible?

### Explicating a social process: year three

In the third year of our study, we will begin the process of mapping the social relations informing care. In many research traditions this is described as “triangulation”. The key difference however between a positivist use of the term “triangulation” and most critical qualitative research approaches is that the purpose of the activity is not to validate a truth claim but rather to extend our understanding of a phenomenon. Starting with insights gained from interviews we will identify “active texts” to discover how these texts are used in the work of primary care physicians and that coordinate this work with the work of others in different settings. We will also be mapping how the consciousness of individuals is coordinated with those of others. For example, how do the discourses of *efficiency, integration* and *patient-centered care* enter the talk of primary care physicians when they are describing their ever yday work caring for complex patients with OA pain? Does these terms map to a higher order text?

### Data management and steps to ensure quality

All interviews will be audiotaped and transcribed. Transcripts will be entered in NVivo software program for data management. It should be noted, however, that while NVivo is useful for managing large qualitative data, it does not perform analysis – this will be undertaken by the researchers. Quality in qualitative research is evaluated differently from the criteria used in quantitative studies. While this is an area of some controversy, a number of strategies will be employed to enhance the trustworthiness of the findings [54–56] including recursive questioning during the interviews and audit trails.

In much research, the researcher’s presence is treated as a bias that must be overcome [52]. As Smith [51] notes, an IE does not rely on notions of objectivity in order to produce “validity”. However, it does strive to produce accounts that are accurate representations of how things actually work. Smith stresses the importance of remaining “faithful to the accounts provided by people of their lived experience while going beyond that experience to explicate how that experience happened as it did” [51]. With this in mind, most qualitative researchers speak of authenticity and reflexivity as central to the goal of “getting it right”. We will make good use of the multi- disciplinary nature of our team to enhance our reflexivity as the diversity of our group will provide many opportunities to challenge our own and others’ assumptions as we proceed through the stages of data collection and analysis.

## Discussion

The “underdevelopment” of relationships between sociology and other disciplines as well as between healthcare organizations has been long noted [10]. Yet there is growing recognition of the potential and need for research that brings together perspectives from a range of disciplines [46]. For example, current knowledge translation frameworks and empirical research in implementation science [34] cite context as the critical consideration in the effective integration of research evidence into practice. However, there remains a dearth of knowledge about how to define, measure, or engage with context [47]. On the other hand, the notion of “situated knowledge” is common amongst many sociological traditions and is well described, used and applied [48]. It is our belief that context and situated knowledge may well be referring to the same phenomenon. In order to empirically study situated knowledge, we may thus borrow from sociological research traditions. Studying the health care institution as it is experienced at the level of people’s everyday work means being able to see it “in motion” [9] and to explore how various texts and competing discourses inform our understanding of the increasingly complex healthcare environment. Through their work, IE researchers are helping to redefine the problems, rethink the questions and design and conduct studies that explicate how things actually work in practice [9].

## Conclusion

Few studies have been able to adequately capture or address the complexity of the care environment in which primary care operates in relation to many other specialties, diseases and diverse patient populations. Our study is novel in that it aims to ground itself in a particular standpoint, not to focus on individual subjective experiences or meaning, but to begin to locate the social coordination of the work of primary care physicians as it is provided in and across both community and academic health care settings. Based on primary care physicians’ responses we will begin to identify the various other provider groups involved in patient care delivery for complex patients, both directly and indirectly, and during a second wave of interviews we will speak to them. This will allow us to identify how the work of coordinating care across multiple settings is accomplished, in practice as well as discursively and textually. Focusing on patients with chronic OA pain will allow us to be specific in our questions, while building on our previous work with chronic pain patients. Further, it renders our study manageable. In a sense, the OA patients will provide the case study for our exploration. However, as in keeping with the exploratory nature of this approach, we will remain attentive and open to physician accounts of other complex patient groups. Our goal is to map an institutional process of providing care that may be similar and therefore useful across many disease groups. Our study is poised to make a significant contribution to our understanding of interdisciplinary, inter- professional and community-based partnerships. An expected outcome of this study will be the development of new, or augmentation of existing, models of care, that are based in the local realities of primary care practice.

## Trial status

We are currently in year one of this project. Key informant interviews have been conducted and transcribed, but have not yet been analyzed.
